# The Inventiveness of Nature: An Interview with Werner Arber

**DOI:** 10.1371/journal.pgen.1004879

**Published:** 2014-12-18

**Authors:** Jane Gitschier

**Affiliations:** Departments of Medicine and Pediatrics and Institute for Human Genetics, University of California San Francisco, San Francisco, California, United States of America

I admit to having a soft spot for the bacterial phenomenon of host-specific modification and restriction. I vividly remember first learning about this mechanism to stave off marauding DNA as a beginning graduate student in the mid-1970s. Preceding the discovery of its modern-day defensive counterpart, CRISPR (clustered regularly interspaced short palindromic repeats) by about 50 years, restriction and modification was not only a remarkable story in itself, but also spawned extremely useful technology. In past issues, I have interviewed Hamilton Smith, who isolated the first type II restriction enzyme, as well as Herb Boyer, whose production of recombinant DNA in collaboration with Stan Cohen launched the biotech industry. For this interview, I was able to go back even farther into the tale and hear first-hand how restriction and modification came to be understood at the molecular level.

This came at the tuition of Werner Arber (Image 1), who received the Nobel Prize together with Smith and the late Dan Nathans. Arber remains active in science; he heads the Pontifical Academy of Sciences and has a keen interest in understanding evolution's molecular drivers, one of which—horizontal gene transfer—is a direct descendent of his work on phage transduction. In early June, after a quick jaunt to Paris (to watch Rafael Nadal win the French Open for the ninth time), I popped over to Basel to reunite with old friends and to interview Arber. Despite that Monday being a Swiss holiday, Arber suggested meeting with me in his office on the top floor of the Biozentrum. It was exceptionally hot, a record-breaking 98°F (37°C) outside, and possibly even hotter indoors. Over three hours, we worked our way through a large bottle of water, which his wife, Antonia, had thoughtfully suggested he bring along. Neither of us flagged as he reminisced about the field and his work in 1950s and ′60s.

Arber developed his interest in natural science as a child growing up on a farm near Aarau, south of the Jura Mountain range of Switzerland. After attending the local gymnasium, he studied at the Swiss Polytechnical School in Zurich to become a science teacher, rather than pursuing his mother's hopes for his priesthood in the Protestant church. He majored in experimental physics and completed his undergraduate degree in 1953, just after the structure of DNA was discovered, and went on to the University of Geneva for his PhD. We pick up the conversation at this transition.

**Gitschier**: You finished your teaching degree, but you were thinking about doing basic research?

**Arber**: Yes. Most of the physicists did not take the broad curriculum I had, including biology and so on. So Paul Scherrer [the prominent Swiss physicist who was Arber's advisor] suggested I go to the University of Geneva where they had an electron microscope, which needed daily care by a physicist. There they looked at a variety of biological objects: bacterial extracts, viruses, bacteriophages, and so on. Right away he phoned Edouard Kellenberger, who ran the Laboratoire de Biophysique in the basement of the Institute of Physics there. It housed electron microscopy as well as radiation studies. I went there within the next few days, and I was satisfied and accepted the job.

I have to tell you the beginnings of this. The Institute of Physics had been headed by another Swiss professor, Jean Weigle. Weigle had agreed to help a Swiss company specialized in vacuum systems to develop an electrostatic electron microscope. It was given to the Institute in the 1940s, free of charge.

Weigle then had some health problems and later quit the directorship of Physics. He was very interested in biology, and he moved to Caltech in California in 1949. But he still had an apartment in the old town of Geneva, and he came to Geneva every summer for three months. At Caltech, he worked with Max Delbrück; he was a part of that relatively small, at that moment, phage group.

**Gitschier**: Presumably, he knew Delbrück through the physics connection.

**Arber**: Absolutely. He brought to Geneva the connection to the phage group, as well as microbial strains. He normally stopped over in Paris at the Pasteur Institute where [François] Jacob and [Jacques] Monod were working, and we had, therefore, a very good connection with these microbial geneticists.

Weigle also had bacteriophage lambda, which originally had been discovered by Esther Lederberg as prophage in *E coli* K12 bacteria.

The problem was that everybody had K12, which was lysogenic, but they didn′t have lambda-sensitive, nonimmune strains. And Weigle brought us both from Esther—the lysogenic and the sensitive strains. In fact, that was not easy because Esther said she wanted to do some experiments with that phage herself. But Weigle said, “We have an electron microscope and we can look at it,” and everybody was interested to see what it looked like! And that was what Kellenberger did—he pleased the involved scientists with a nice electron micrograph of phage lambda.

**Gitschier**: And were there other types of bacteriophage that they looked at?

**Arber**: Of course, the T-evens and a number of other phages. One of my jobs in the lab was to look at mutant bacteriophages; there were about ten different mutants of lambda available. These were prophages, so I could induce by UV irradiation and then look at the lysate. Kellenberger was very good at inventing new preparation techniques. You could spread the lysate out on the film and see and count phage-related structures. In nonmutant strains of lambda, of course, there were the nice phages with heads and tails, whereas for some of the mutants, there were heads but no tails attached, and sometimes the tail was missing. In some other cases the heads were empty.

**Gitschier**: Where did all these mutants come from?

**Arber**: From the lab itself, and we got some from the Pasteur Institute. Then one day, Weigle brought me the defective lambda transducing prophage that had been isolated in the Lederbergs' lab by Larry Morse. He asked whether lambda could also transduce, like P22, and it did! But it transduced only galactose markers. And that was only upon induction of phage production by lysogenic bacteria, not after lytic infection. Now we know that the attachment site for the lambda prophage is near the galactose markers…

**Gitschier**: … in the *E. coli* genome.

**Arber**: Yes, and at frequencies of 10^−5^, the lambda genome crosses out imprecisely from its attachment site to form lambda-gal, but this hadn′t been known yet at that time. So I induced the defective lysogen that we received from Weigle with UV irradiation, and the cells lysed but I couldn′t see any phage-related structures in the lysate.

**Gitschier**: No phage particles?

**Arber**: Indeed, no phage particles, no empty heads, no tails!

At that moment, I decided to start genetic experiments. There were a certain number of already mapped mutants available—for head, tail, and other properties. From induction of double lysogens— having one lambda-gal and one active phage—you get both active and transducing particles. In a series of genetic experiments, I could show that in the lambda-gal genome, a few head and a few tail genes were just missing. Then we realized that instead of having these head and tail genes, they must carry gal genes. That was one of the early clear proofs that some viruses can serve as vectors for host DNA integrated into the viral genome. This is now called specialized transduction. I reported our findings at the Assembly of the Swiss Society for Microbiology. A few people may have understood it.

**Gitschier**: What year?

**Arber**: That was in 1957. With the impulsion of Edouard, I was invited to a conference of microbial geneticists at Royaumont near Paris, to present my work, and that had a really big echo. On the spot I got two invitations for post-doc—one by Joe Bertani in Los Angeles, and the other by François Jacob at the Pasteur Institute. I thought both would be interesting, but I could only accept one; I chose the United States rather than France. A few years before I came to Bertani's lab, he had hoped to obtain phage lambda and appropriate hosts from the Lederbergs, but this was apparently quite difficult. He therefore decided to isolate phage strains from other lysogenic bacteria himself. He called these phages P1, P2, and P3.

Joe and his wife Betty decided to work with P2, and they did a lot of experiments with P2 using various hosts. P1 is a transducing phage, and Joe told me I could do anything I wanted. Practically every day we went to the cafeteria to have lunch together, and we spoke on science. At the end of that one-year period, I had enough data to draft two papers. I showed them to Bertani—I had put his name as coauthor—and he said, “These are nice papers but you are the author.” I got so much input from him every day, but I had to accept his opinion. I submitted them to *Virology*; Salvador Luria was one of the editors. He accepted both papers, and at that moment he offered me a second post-doc. I also got invited by Joshua Lederberg to work with his wife Esther at Stanford, and I got a third offer from Edouard Kellenberger to return to Geneva!

After one year of post-doctoral work in Los Angeles, I left Bertani's lab and went to Gunther Stent in Berkeley for a few weeks, because I wanted to learn the “suicide” method that he had invented with very heavily ^32^P-labeled phage. You grow the phage in ^32^P medium, store them, and assay the survivors every few days. After that stage, I went to Stanford to work with Esther for one month, and then on the way back to Geneva I stopped over at MIT to work for another month with Luria.

**Gitschier**: So tell me, while we are on the subject of Esther. Was lambda the first phage to be discovered to have this property of lysogeny?

**Arber**: No. Lysogeny was a known phenomenon, and actually André Lwoff had explored lysogeny with other types of lysogens.

**Gitschier**: So, what was it that was so special about finding lambda?

**Arber**: From my point of view, the special thing was specialized transduction. A short background: conjugation had been shown to work with *E. coli* K12. And, in fact, another student of the Lederbergs was Norton Zinder who was charged with looking for conjugation in *Salmonella* strains. And at first view, it seemed that there was recombination. But then they could show that there were no cell–cell contacts. In the conjugation mixture some of the cells lysed, being lysogenic for phage P22, which turned out to be a transducing phage. That was the discovery of transduction by Zinder.

And then Larry Morse was charged with investigating whether lambda also does that. And it did—but only for galactose, while the *Salmonella* phage P22 gives generalized transduction, and so does phage P1. These phages transduce any host genes.

**Gitschier**: So, lambda was interesting because it was different.

**Arber**: Yes. And with my observation of the genetics, it became clear how, in specialized transduction, some host genes can become part of the viral replicating unit. The defective phage lambda-gal still undergoes replication, but it doesn′t make functional coats and tails. In contrast, there is no trace of viral DNA in transducing P1 and P22 particles. In generalized transduction the infective transducing phage particles are just filled with a segment of host DNA.

**Gitschier**: Got it!

**Arber**: From bacterial conjugation experiments, it had become known by the late 1950s that DNA are very long filamentous molecules carrying their genetic information in a linear order. As a matter of fact, the conjugation results suggested that the *E. coli* chromosome is only one circular molecule.

This raises the question of how can one best study single genes, one by one. People then proposed to sort out short genomic segments and to splice them into a natural gene vector such as the lambda genome. Because the hybrids are replicative units, they can be expected to replicate the inserted gene separately from the rest of the bacterial genome. This can yield enough material to undertake both structural and functional analyses.

**Gitschier**: Wow! So people were thinking ahead to sequence analysis even back then?

**Arber**: Yes, right! People were thinking just to break the DNA by sheering forces, but they didn′t get very far with that. So the idea of recombinant DNA was already there, but one didn′t know how to do it best.

**Gitschier**: Now I understand the significance of lambda.

**Arber**: I have to tell you now about the offer I got to return back to Geneva. This was in 1959. In physics, they intended to develop atomic power plants in order to get a new energy supply. Paul Scherrer managed to convince the Swiss Parliament to vote for a big financial credit just for that. And Edouard had made a request that a very small part of that big credit should be spent to study radiation effects: biosafety. He got that money, and he asked whether I would come back to lead that group. So I went back to Geneva and I planned to study radiation effects on bacteria and on phages.

**Gitschier**: What kind of radiation?

**Arber**: Any radiation. Starting with UV, which we used for induction of phage production by lysogenic bacteria, X-ray, and then also I intended to incorporate heavy radioactivity, ^32^P, in the DNA. That's why I went to learn the “suicide” technique from Gunther Stent.

**Gitschier**: So, you were already planning to come back here.

**Arber**: Right. And the other thing I had known was that Evelyn Witkin had isolated radiation-resistant mutants from *E. coli* B. The strain was B/r, and it was already available in several labs. [Readers may want to refer to the Witkin interview in 2012.] And of course, B is not a host for lambda, because lambda does not adsorb to B.

**Gitschier**: So, you had a problem because you wanted to study lambda in the radiation-resistant strain.

**Arber**: Right. But Esther Lederberg gave me a hint; she knew that *E. coli* B is maltose minus, and she thought that the maltose minus character is responsible for not adsorbing lambda.

**Gitschier**: Because the maltose transporter might serve as the lambda receptor?

**Arber**: Right. So she suggested to transduce *E. coli* B with P1 grown on K12, and to select for maltose-plus derivatives. And she was right. About one of three of the maltose-plus transductants adsorbed lambda.

**Gitschier**: Wow!

**Arber**: But then, my lambda preparation came from K12, and it adsorbed to the transductant B/r, but almost no phage was produced.

**Gitschier**: So this is a multistep process. After all of this manipulation, only a few progeny phage came out if you′ve grown them before in K12. So B restricts lambda that is grown in K12.

**Arber**: Precisely. And those few lambda that come out of B can go back to B without restriction. Weigle and Bertani had already worked on host-controlled modification in both, P2 and lambda, and I was fully aware of their work. This had happened maybe five years before.

**Gitschier**: I was wondering why Bertani and Weigle didn′t keep working on that?

**Arber**: I don′t know.

**Gitschier**: So now we are at the point that you tried to take the lambda from K12 and grow it in B/r mal^+^ and you realize that you have host modification problems.

**Arber**: Right. Grete Kellenberger had done already some studies on radiation effects on lambda and shown that, when heavily UV-irradiated, DNA becomes rapidly acid-solubilized, which means it is degraded; same thing with ^32^P heavy labeling. So they wondered if upon restriction, the DNA is also degraded and solubilized.

When I came back to Geneva, there was a student starting her PhD study in the radiation field: Daisy Dussoix. She started to work with me on that question, and one of the first experiments was to see whether, like for irradiated phage, restricted phage DNA was also degraded. And it was.

**Gitschier**: And did you do that with ^32^P labeling?

**Arber**: Yes, but not the heavy one, just slightly labeled, as a tracer.

So then, I felt that we should also study the restriction modification mechanisms in more detail. Now, retrospectively, we know that the reasons for DNA degradation are different between irradiation and restriction. But actually, the money came from the nuclear power plant funding to study radiation effects, so I had to justify why we did that other project on host-controlled modification.

But before I justified it, we did a very important experiment. If you look at our first *JMB* [*Journal of Molecular Biology*] paper on restriction and modification in 1962, you′ll be surprised that I did it with the “suicide” technique. Of course, I had read about semiconservative DNA replication by Matt Meselson and Frank Stahl, so I knew the principles of DNA replication.

**Gitschier**: That was ′58.

**Arber**: Yeah, and my experiment was done in 1960. I prepared a stock of lambda, which was heavily loaded with ^32^P—“suicide” levels. Immediately after its preparation, the phage stock was carefully purified from the radioactive medium and then used for a one-cycle growth in a nonmodifying host in nonradioactive medium.

Taking into account the semiconservative DNA replication, I expected that in the one-cycle lysate obtained after very low multiplicity of infection, there would be no conserved DNA, but at most, two viruses with semiconserved DNA per lysed cell, whereas all the other phage genomes would be new, without ^32^P. And, in fact, that was confirmed. Only parental DNA, which was still subject to radioactive decay, actually had the parental modification. Most of these experiments were done with P1 restriction [phage P1 carries its own modification and restriction system], but I did it later also with B and with K12 hosts.

**Gitschier**: So at this stage, you know it is something about the DNA.

**Arber**: That was indeed new! One did not expect it would be about the DNA.

**Gitschier**: These people prior to you—Luria and Bertani—they are all saying it's not a mutation, it's a modification. Was there anything known at the time about methylation?

**Arber**: OK, I′ll tell you. I knew Gunther Stent very well, and one day Stent said they had a fellow who worked with nucleic acid methylation.

**Gitschier**: Oh, really? Was DNA known to be methylated at that time?

**Arber**: There were publications on DNA methylation in eukaryotes. And some of these papers postulated that DNA methylation might have something to do with cancer.

**Gitschier**: But not yet in bacteria.

**Arber**: No. But Stent wondered if bacterial modification is, in fact, DNA methylation for which methionine would be the methyl donor. I happened to have methionine minus bacterial hosts. You grow them in the presence of methionine. These bacteria are then infected with the phage, or in the case of lysogenic cells, phage reproduction is induced by UV irradiation. After a few minutes of further incubation in the complete medium, the cells are washed and then further incubated in medium without methionine. Under these conditions, the majority of the produced phage was not modified, even though the bacterial host was a modifying strain.

**Gitschier**: The specific testing of methylation doesn′t come across in your 1965 paper [one of a series of *JMB* papers on host specificity]. You test several auxotrophs, but you don′t say you are testing methionine with the others as controls. At the very end of the paper you say that the chemical nature is unknown, but that alkylation is an attractive possibility.

**Arber**: At that time I considered the experiment with the methionine-minus host as an indication, but not as a proof that modification occurs by DNA methylation. In the same time period, I was invited to write a contribution to *Annual Review of Microbiology* [published in 1965], and there I speak about methylation as a likely basis for modification.

**Gitschier**: So, just to be clear, this host modification process was really discovered as a phage process. And later it was understood to be a basic problem.

**Arber**: I realized rather rapidly that this was not limited to phage. And, of course, we could then show that it also affects conjugation, DNA transformation, and transduction. It works in any of these processes. We did all these experiments to show that it is a general phenomenon of the host.

Actually, the proof for methylation came later on when some of my collaborators succeeded in isolating the modification enzyme. This enabled us to show that the modifying enzyme is a methylation enzyme. I, myself, was not feeling like doing enzymology, but I had very good collaborators, like the Americans Bill Wood and Stuart Linn, as well as Urs Kühnlein, a Swiss PhD student.

**Gitschier**: It was in the late ′60s before the restriction enzymes were isolated.

**Arber**: Yes. Matt Meselson had moved to Harvard, and Bob Yuan was one of his young collaborators. In 1968, they were the first to isolate the K restriction enzyme, and to determine its cofactor requirements, on which our group had been properly informed.

**Gitschier**: This is a type I enzyme that just chops things up all over the place. But the methylation, even there, is at specific sites.

**Arber**: Yes! There are recognition sites. This was always my idea that there were specificity sites. I had expected that the enzymes would generally be of type II, i.e., specific cutting sites.

**Gitschier**: But you were able to show that the methylation was happening in specific places in the DNA?

**Arber**: For a long time, that was a hypothesis.

**Gitschier**: Because I was wondering, without type II restriction enzymes, themselves, to specifically cleave the DNA, how can you even figure that out?

**Arber**: I called on John Smith, an enzymologist from England, and he spent about half a year with us in Geneva. He was quite successful in mapping the sites of methylation. They had mastered RNA sequencing, but not yet DNA sequencing, so it was hard work to determine the recognition sites.

From today's knowledge on type I's, let's say you have a recognition site here, here is another one, here still another one; if unmodified DNA molecules come into the restricting cell, then the enzyme identifies, first, one of the specificity sites which has no methyl group. But it does not cleave right away. It starts to rope through the DNA on both sides. This can be shown by electron microscopy. Meanwhile, another enzyme molecule identifies another specificity sequence and starts to rope through the DNA. Sooner or later, the two enzyme complexes run into each other. That stops the translocation and initiates the cleavage of the DNA molecule.

**Gitschier**: Oh, fascinating!

**Arber**: This functioning has been shown with small DNA molecules, and it is indeed wonderful. In the presence of the type I enzyme, each time you bring in a foreign DNA, it gets cut at a different place because the recognitions at different specificity sites are not synchronous. So sometimes you cut here, sometimes you cut there. And that means that all the fragments are *different* and you do not always kill a particular gene that might be useful to the receiving cell's progeny. In type II, you always cut reproducibly at the recognition site, and if that is a part of an important gene, you kill it by restriction!

So, for evolution involving steps by horizontal gene transfer, the type I's are actually more appropriate than type II enzymes. And for me, this is fantastic.

**Gitschier**: How are you feeling? Do you have energy for another fifteen minutes or so?

**Arber**: No problem. I′m surprised and satisfied to see how informed you are.

**Gitschier**: Thank you! So let's briefly delve into this horizontal gene transfer a bit more.

**Arber**: OK, I can just tell you how I came into studying molecular Darwinism. I was an EMBO member already in Geneva, and after moving to the University of Basel, I proposed to organize an EMBO workshop near Basel, at a religious meeting place called Leuenberg. This international workshop on bacterial restriction and modification was held in the fall of 1972. Ham Smith and Herb Boyer were there, and many other scientists who had contributed to the field. This was the time when recombinant DNA work had successfully started, thanks to the availability of restriction endonucleases. A few American participants raised the question whether there were some conjectural risks associated with the recombinant DNA technology. So we spontaneously inserted into our program an evening session to talk about these potential risks.

**Gitschier**: This is well before the Asilomar Conference.

**Arber**: Yes. And since we also wanted to include ethical aspects, we invited the protestant priest who managed the meeting center to attend our debate. We did not end up with clear conclusions, but we identified relevant issues and questions deserving more profound attention. Some of the participants of this evening discussion were later among the scientists signing a letter to *Science* in 1974 requesting an international conference on risk assessment for recombinant DNA technology.

That conference was held in February 1975 in Asilomar. Its participants suggested distinguishing between short-term and long-term risks. Short-term risks are those for people who work in the laboratory: a cloned gene with yet unknown function could be toxic or pathogenic, so one should be careful and take the same precautions as for medical microbiological analyses when samples from patients are studied. But long-term risks could relate to the release of recombinant DNA into the environment. Then, one cannot know whether it can later spread horizontally. In the 1970s, horizontal gene transfer was well known for microorganisms, but most eukaryotic people claimed that horizontal gene transfer does not concern higher organisms.

**Gitschier**: That's because they didn′t know enough.

**Arber**: Indeed. Then I, and a few other people, realized in Asilomar that the time had come to study the molecular processes in Darwinian evolution. In doing so with microbial populations, I came to realize that nature is quite inventive.

There are a multitude of specific mechanisms at work to spontaneously promote occasional genetic alterations. I classified them into three natural strategies of genetic variation. One of them promotes local nucleotide changes upon DNA replication: a nucleotide substitution, the insertion or deletion of one or a few nucleotides, or a mingling of a few nucleotides. By chance, these processes can affect an existing function either positively or negatively.

The second strategy is a rearrangement of DNA segments within the genome, and it is a source for deletion, amplification, inversion, or translocation of a DNA segment. These processes are usually driven by specific enzymes. Phage P1, for example, carries a gene called *cin* (for C-inversion). Under its action, the C-segment of the P1 genome gets periodically inverted between two specific crossover sites. This gives rise to a flip-flop process affecting a tail fiber gene, which determines the host range of the phage. We have seen that, at rare occasions, the *cin* gene product can also use a number of secondary crossover sites. This kind of fusion can, by chance, result in a novel gene activity or in an alternative efficiency of expression of a concerned gene.

The third strategy of genetic variation is horizontal gene transfer, i.e., the acquisition of a short segment of genetic information from another kind of organism. This may sometimes happen upon cohabitation of different kinds of organisms.

**Gitschier**: I recall the initial intrigue when the human genome was sequenced, that there were something like 150 genes that were suggested to come from bacteria. This hasn′t panned out, but there are other examples, like integration of *Wolbachia* [an intracellular bacterium] genes into the genomes of some insects.

**Arber**: Nature is much more complex than we believe. I don′t think there is much work so far on horizontal gene transfer between higher eukaryotes and microorganisms. It is always true that you can get new insights if you develop new methodologies. This will come.

**Figure:**
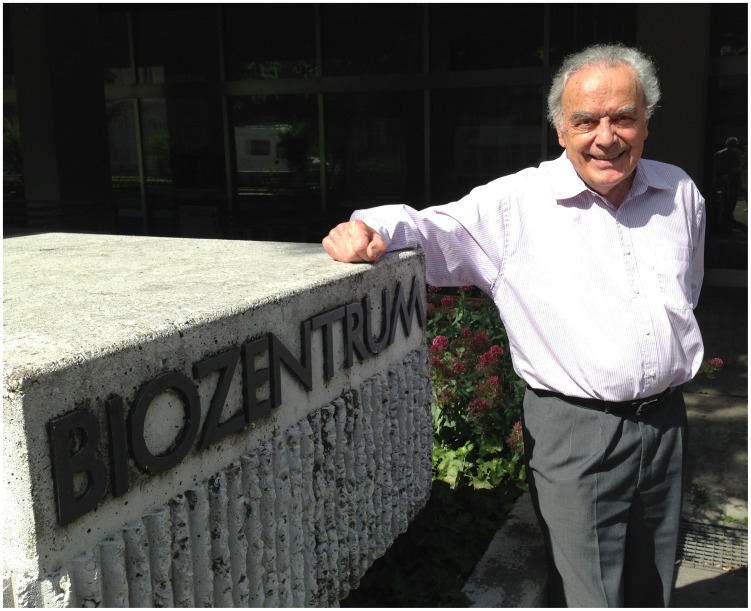
Werner Arber stands outside the Biozentrum at the University of Basel, Switzerland.

